# Unusual presentation of peritonitis with persistent clear aspirate: a case report

**DOI:** 10.1186/1752-1947-4-383

**Published:** 2010-11-28

**Authors:** Ebru Asicioglu, Arzu Kahveci, Elif Ari Bakir, Atilla Bulur, Hakki Arikan, Mehmet Koc, Serhan Tuglular, Cetin Ozener

**Affiliations:** 1 Department of Nephrology, Marmara University School of Medicine, (Tophanelioglu Cad.), İstanbul, (34660), Turkey; 2 Department of Internal Medicine, Marmara University School of Medicine, (Tophanelioglu Cad.), İstanbul, (34660), Turkey

## Abstract

**Introduction:**

Peritonitis is the most frequent complication of peritoneal dialysis. Diagnosis of peritonitis includes symptoms and signs of peritonitis with a cloudy aspirate of more than 100 WBC/ml, as well as positive cultures. Although sterile peritonitis has been reported in the literature, to the best of our knowledge this is the first report of an unusual presentation of peritonitis without any white blood cells in the peritoneal aspirate despite multiple positive peritoneal cultures.

**Case presentation:**

An 82-year-old Caucasian man who had been on continuous cycling peritoneal dialysis for 12 years was admitted to our hospital with general malaise, loss of appetite, weight loss and somnolence. He did not describe abdominal pain or fever. Even though his peritoneal fluid was consistently negative for leukocytes and clear, he had peritonitis with different organisms consecutively.

**Conclusions:**

Our case report shows that any patient on peritoneal dialysis presenting with evidence of infection (fever, peripheral leukocytosis) without an obvious cause should have aspirate cultures done even if the aspirate is clear and abdominal pain is absent. Our case report may change the initial work-up and management of these patients. We believe this report is of interest to general medicine and emergency room physicians as well as nephrologists.

## Introduction

Peritonitis is the most frequent complication of peritoneal dialysis (PD). Diagnosis of peritonitis includes symptoms and signs of peritonitis with a cloudy aspirate of more than 100 WBC/ml, as well as positive cultures. Although sterile peritonitis has been reported in the literature [1], to the best of our knowledge this is the first report of an unusual presentation of peritonitis without any white blood cells in the peritoneal aspirate despite multiple positive peritoneal cultures.

## Case presentation

An 82-year-old Caucasian man who had been on continuous cycling peritoneal dialysis (CCPD) for 12 years was admitted to our hospital with general malaise, loss of appetite, weight loss and somnolence. He did not describe abdominal pain or fever. His medical history included renal failure due to nephrolithiasis and he had not experienced peritonitis before. His physical examination was unremarkable and he was experiencing no abdominal tenderness at the time of his admission. His white blood cell count was 9600/ml with 80 percent neutrophils. His C-reactive protein (CRP) level was 118 mg/L (Normal range: 0-5 mg/L). His blood cultures were negative. He was started on ampicillin and sulbactam and ciprofloxacin treatment empirically. Even though his peritoneal fluid was clear and there were not any leukocytes present, peritoneal fluid cultures taken on admission revealed *Pseudomonas aeruginosa*. The ampicillin and sulbactam was switched to piperacillin and tazobactam and ciprofloxacin treatment was continued since the isolate was sensitive to both. Two weeks after his admission, he developed a cough with sputum production. There was a new infiltration on his chest X-ray consistent with pneumonia and his CRP level increased from 32 mg/L to 152 mg/L. He was switched to imipenem and vancomycin therapy. He still did not describe abdominal pain or fever and there was no abdominal tenderness on physical examination. Even though the peritoneal fluid count did not show any leukocytes, a control peritoneal fluid culture revealed *Stenotrophomonas maltophilia *which was sensitive to trimethoprim-sulfamethoxazole. Trimethoprim-sulfamethoxazole was added to treatment. Despite treatment with imipenem, vancomycin and trimethoprim-sulfamethoxazole for two weeks, his condition deteriorated rapidly and he developed septic shock. While analysis of his peritoneal fluid was still negative for white blood cells, peritoneal cultures obtained during the septic shock revealed vancomycin-resistant *Enterococcus faecium *(VRE). His blood cultures were negative, however stool cultures also revealed VRE. He was started on linezolide therapy however his condition deteriorated rapidly and he died six weeks after hospitalization.

## Discussion

Peritonitis causes significant morbidity and mortality in PD patients. It can result in death either from sepsis or from ensuing complications. Gram-positive organisms, especially *Staphylococcus epidermidis *and *Staphylococcus aureus*, are the most common bacteriologic causes of peritonitis. Recently, the incidence of Gram-positive infections has decreased due to newer techniques and improved exit-site care, while the incidence of Gram-negative organisms has remained stable [2]. Gram-negative organisms now account for 20 to 30 percent of all peritonitis episodes [3]. Patients with Gram-negative peritonitis have a worse outcome, are more likely to be hospitalized and more likely to die within six months after peritonitis [2]. In our case report, peritoneal fluid cultures yielded three different organisms consecutively. The first organism was *P. aeruginosa *which is one of the most important causes of Gram-negative peritonitis and is identified in 7.1 percent of culture positive cases [3]. The major risk factors for infection are a history of antibiotic therapy within 30 days and concomitant exit-site infection [3]. Our patient did not have any risk factors for this infection. The second microorganism was *Stenotrophomonas maltophilia *which is an environmental saprophyte that has been rarely reported to cause peritonitis. The annual rate of isolation for this organism is not known since it has only been reported as case reports. It is mainly a nosocomial pathogen and is resistant to multiple antibiotics [4]. It can be isolated from water, soil, hospital equipment and humans. The major risk factors include prior broad-spectrum antibiotic use, neutropenia, underlying illness and prolonged hospital stay [4]. The patient described in this case report had risk factors for this organism, such as prolonged antibiotic use and hospital stay. The third microorganism was VRE, which was first described in the 1980 s and has since become increasingly prevalent, causing infections that include PD-associated peritonitis [5]. However, the annual rate of isolation is not known. Risk factors for VRE include severe underlying disease, immunosuppression, prior use of vancomycin and other antibiotics, abdominal or cardiothoracic surgery and indwelling urinary or central venous catheters [6]. Patient had multiple risk factors for VRE infection, such as vancomycin use and prolonged hospital stay. The persistent growth of organisms in his peritoneal culture and his lack of response to antibiotic therapy warranted early removal of the catheter. Failure to respond to antibiotic therapy necessitating early catheter removal is common in patients with *S. maltophilia *and/or VRE peritonitis. Our patient was repeatedly advised immediate surgical removal of his PD catheter due to the risk of septic shock and death; however, he refused to undergo the procedure despite regular counseling

The majority of patients with peritonitis have cloudy fluid and abdominal pain. The diagnosis is confirmed by a dialysate aspirate leukocyte count exceeding 100 WBC/ml with at least 50 percent polymorphonuclear leukocytes. A small percentage of patients do not have white blood cells in the peritoneal fluid at presentation with cell counts increasing in time; this is called an impaired initial cell reaction [7]. This delay may be due to slower cytokine response to infection [7]. Peritonitis episodes with cell counts less than 100 WBC/ml have been reported in the literature [8,9]. However, peritonitis without abdominal pain and/or white blood cells in the peritoneal fluid is an extremely rare entity and to the best of our knowledge, this is the first report of a case of peritonitis with a persistently clear aspirate without any white blood cells. The peritoneal fluid cultures of our case report yielded different organisms consecutively, however the peritoneal cell counts were consistently nil. It can be argued that the positive cultures in the absence of increased white blood cell count in the peritoneal fluid could be indicative of colonization or improper culture collection. The method used for peritoneal culture at our center is as follows: sediment obtained after centrifugation of 100 ml of fluid is inoculated into aerobic and anaerobic Bactec bottles, on aerobic and anaerobic blood, chocolate and MacConkey agar plates. Gram and Giemsa stains are also performed. It is highly unlikely that the only three improper cultures collected at our center so far have been in the same patient. A low white blood cell count in peritoneal fluid in the presence of antibiotic use has been reported. However, since the first organism was isolated before the use of any antibiotic therapy and the consecutive organisms were both resistant to the antibiotic therapy administered at that time, it is not possible to explain the absence of white blood cells by prior therapy. Immunosuppression is another possible mechanism for the lack of white blood cells in our case report; however, he did not have any disease, condition or any other laboratory values indicating that he was indeed immunosuppressed.

We believe our case report demonstrates that clear aspirate and the absence of white blood cells in the peritoneal fluid may not rule out the presence of peritoneal infection in patients on PD. Therefore, any patient on PD presenting with evidence of infection (fever, peripheral leukocytosis) without an obvious cause should have aspirate cultures done even if the aspirate is clear and abdominal pain is absent.

## Conclusions

Patients on PD presenting with evidence of infection (fever, peripheral leukocytosis) without an obvious cause should have aspirate cultures done even if the aspirate is clear and abdominal pain is absent.

## Consent

Written informed consent was obtained from the patient's next of kin for publication of this case report and any accompanying images. A copy of the written consent is available for review by the Editor-in-Chief of this journal.

## Competing interests

The authors declare that they have no competing interests.

## Authors' contributions

EA, AK, EAB, AB and HA made substantial contributions to the design, and the acquisition and interpretation of data. MK, ST and CO revised the manuscript critically for important intellectual content. All authors read and approved the final manuscript.
